# Coupling p+n Field-Effect Transistor Circuits for Low Concentration Methane Gas Detection

**DOI:** 10.3390/s18030787

**Published:** 2018-03-06

**Authors:** Xinyuan Zhou, Liping Yang, Yuzhi Bian, Xiang Ma, Ning Han, Yunfa Chen

**Affiliations:** 1 State Key Laboratory of Multiphase Complex Systems, Institute of Process Engineering, Chinese Academy of Sciences, Beijing 100190, China; zhouxinyuan14@mails.ucas.edu.cn (X.Z.); yangliping@ipe.ac.cn (L.Y.); bianyuzhi17@mails.ucas.edu.cn (Y.B.); max.nmzx@aliyun.com (X.M.); 2 University of Chinese Academy of Sciences, No. 19A Yuquan Road, Beijing 100049, China; 3 Center for Excellence in Regional Atmospheric Environment, Institute of Urban Environment, Chinese Academy of Sciences, Xiamen 361021, China

**Keywords:** low concentration methane gas, metal oxide gas sensors, field effect transistors, amplification effect

## Abstract

Nowadays, the detection of low concentration combustible methane gas has attracted great concern. In this paper, a coupling p+n field effect transistor (FET) amplification circuit is designed to detect methane gas. By optimizing the load resistance (*R_L_*), the response to methane of the commercial MP-4 sensor can be magnified ~15 times using this coupling circuit. At the same time, it decreases the limit of detection (LOD) from several hundred ppm to ~10 ppm methane, with the apparent response of 7.0 ± 0.2 and voltage signal of 1.1 ± 0.1 V. This is promising for the detection of trace concentrations of methane gas to avoid an accidental explosion because its lower explosion limit (LEL) is ~5%. The mechanism of this coupling circuit is that the n-type FET firstly generates an output voltage (*V_OUT_*) amplification process caused by the gate voltage-induced resistance change of the FET. Then, the p-type FET continues to amplify the signal based on the previous *V_OUT_* amplification process.

## 1. Introduction

Methane is a highly flammable and explosive gas widely used in domestic and industrial applications. The lower explosion limit (LEL) of methane is known to be approximately 5% [1,2]. Thus, the trace concentration of methane should be detected fast and reliably in the environment to prevent dangerous explosions. Moreover, in environmental safety, extreme demands are placed on the detection of the potential greenhouse gas methane [3,4]. Recently, various methane sensors have been developed on the basis of catalytic combustion [5,6,7], metal oxide semiconductor (MOX) [8,9], infrared spectrum [10,11], gas chromatography [12,13], and optical fiber [14,15]. Su et al. reported that the catalytic combustion MEMS (microelectro-mechanical systems) sensors can detect 4000 ppm methane [5]. Shaalan et al. prepared the Co_3_O_4_ nanoparticles with response (*Ra*/*Rg*) of ~1.03 to 2500 ppm methane [8]. The tunable multi-mode diode laser absorption spectroscopy was employed by Gao et al. to respond to 25 ppm methane, with a relative accuracy of 0.27% [11]. There are still challenges in detecting the trace concentration methane. Therein, the MOX gas sensor enjoys a tremendous advantage due to a fast response, chemical stability and low cost [16,17,18].

Great quantities of new sensing technologies have been developed to improve the gas response of MOX gas sensors and the p+n structure sensor is among them. It consists of two sensitive bodies A and B, where A is a p-type sensing material and B is an n-type sensing material. The theoretical calculation showed that the sensor realized the multiplication of sensitivity and selectivity when the resistance of B in air is much less than that of A. Wang et al. have developed a p+n combined ethanol sensor to realize the multiplication of selectivity and sensitivity, as compared with the single p-type or n-type gas sensor [19]. However, it has not been widely applied until now, due to a lack of highly sensitive p-type MOX sensors.

In our previous work, an n-type field effect transistor (FET) circuit [20] has been reported to amplify the voltage signal of the MOX gas sensor. For example, this n-type FET circuit is capable of magnifying the response of TGS2602 sensors to toluene by 5–6 times, so that TGS2602 sensors are able to respond effectively to ~0.1 ppm toluene, lower than the highest permissive limit (0.26 ppm) of toluene in the indoor air of cars. The amplification effect is because there is a dramatic resistance change of n-FET induced by a tiny change of the gate voltage. However, a new circuit with even larger magnification factors (MF) is expected to improve the gas response of MOX sensors, in order to meet the needs of low concentration dangerous gas detection. In this study, both p-type and n-type FETs have been combined in a circuit to form the coupling p+n FET circuit, in order to detect low concentration methane, on the basis of the commercial sensor MP-4.

## 2. Design Scheme of the Amplification Circuit

**Figure 1:**
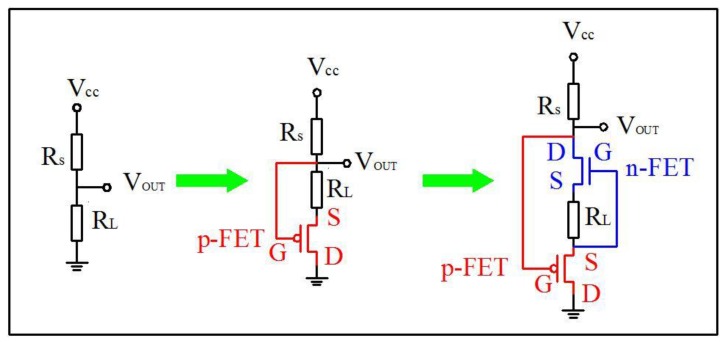
Design scheme of the traditional circuit, the p-type field effect transistor (FET) circuit and the coupling p+n FET circuit for metal oxide semiconductor (MOX) methane sensors.

Figure 1 schematically shows the circuit for the methane sensor, which transfers from the traditional circuit to the p-type FET circuit and then to the coupling p+n FET circuit. For the traditional circuit, load resistance (*R_L_*) and MOX gas sensor (*R_S_*) are put in series in the circuit. Then, a bias voltage of 5 V (*V_CC_*) is applied and the partial voltage of the *R_L_* is the output voltage (*V_OUT_*) signal. Afterwards, select an appropriate *R_L_* to make *V_OUT_* as low as ~0.4 V in clean air. When *R_S_* makes contact with the target gas, the reduced *R_S_* will lead to the increase of the circuit current and further, *V_OUT_* will become larger. Thus, the change of *V_OUT_* only depends on the change of *R_S_*.

The p-type FET circuit is different from the traditional circuit, in that a p-type FET is added to the circuit with source (*S*) connected to *R_L_* and drain (*D*) grounded. According to the *I_D_*–*V_GS_* curve of the p-type FET (seen in Figure S1a), a positive gate source voltage (*V_GS_*) is necessary. Therefore, the gate (*G*) of the p-type FET is connected to the higher potential side of *R*_L_. To make a comparison, the same R_L_ above is chosen. The baseline of the p-type FET circuit in clean air is similar to that of the traditional circuit because of the negligible resistance of the p-type FET (*R_FET_* is ~10^2^ Ω in the ON state). Injecting the target gas, *R_S_* will decrease and thus, the circuit current will increase and so does *V_GS_* of the p-type FET. *V_GS_* shifts positively to let the FET work in the OFF state (*R_FET_* is ~10^9^ Ω), which leads to much larger *V_OUT_*. In all, *V_OUT_* is under the double influence of *R_S_* and *R_FET_*.

Subsequently, an n-type FET is inserted between *G* and *S* of the p-type FET forming a coupling p+n FET circuit, as shown in Figure 1. Similarly, *V_OUT_* of this coupling circuit in clean air is roughly as low as that of the former two circuits above because both p-type and n-type FETs are in the ON state. When the target gas is injected, *R_S_* will fall, resulting in the rise of circuit current and the consequently *V_GS_* negative shift of n-FET (seen in Figure S1b). Since the resistance of n-FET is more sensitive to the change of *V_GS_* (seen in Figure S1c), the negative shift *V_GS_* induces the moderate increase of the FET resistance, followed by the *V_DS_* growth of n-FET, which is the first *V_OUT_* amplification process. Driven by the increasing *V_DS_* of n-FET and the partial voltage of *R_L_*, *V_GS_* of p-FET shows a clearly positive shift inducing the resistance of p-FET to rise by several orders of magnitude, with the second *V_OUT_* amplification process occurring. In brief, the single p-type FET circuit generates the *V_OUT_* amplification process only once, while this coupling circuit does twice.

## 3. Experimental

The methane gas sensors (MP-4) were purchased from the market. The transfer curves (*I_DS_–V_GS_*) of typical n-type FETs and p-type FETs are measured by the Keithley 4200 semiconductor analyzer shown in Figure S1, where both 2SK184 and 2SK364 show larger *I_DS_–V_GS_* slopes (the smallest subthreshold swings), when compared with other n-type FETs and so do both 2SJ44 and 2SJ45 FETs. The larger slope makes the FET more sensitive to the gate voltage change, leading to the larger amplification effect according to our previous work [20]. Thus, FETs (2SK184, 2SK364, 2SJ44 and 2SJ45) are utilized to form the coupling p+n FET circuit. All of them are used without any modification. The static gas sensing test system (Hanwei WS-30A, Zhengzhou, China) [21,22,23,24] is utilized to study the sensing property of MP-4. The load resistance card is the standard accessory of the system and p-FETs and n-FETs are soldered orderly onto the resistance card with D, S, and G electrodes shown in Figure 1. Inject a different volume of 5% standard methane gas into the gas chamber (total volume 18 L) to generate a different concentration of methane gas, varying from 10 ppm to 150 ppm. *Ra* and *Rg* are sensor resistances (*R_S_*) in air and methane gas respectively. Thus, the response of MP-4 to methane is defined as Response = *Ra/Rg* [25,26,27] for the traditional circuit without FET, while the response is defined as Response = *Ra/Rg* × *MF* for the amplification circuit with FET. The magnification factor (*MF*) of FET is defined as *MF* = (*R_L_* + *R_FET_*_,__g_)/(*R_L_ + R_FET_*_,__a_) [20], where *R_FET,__a_* and *R_FET,__g_* represent FET resistance in the air and methane gas respectively.

## 4. Results and Discussion

### 4.1. Single FET Amplification Circuit

**Figure 2:**
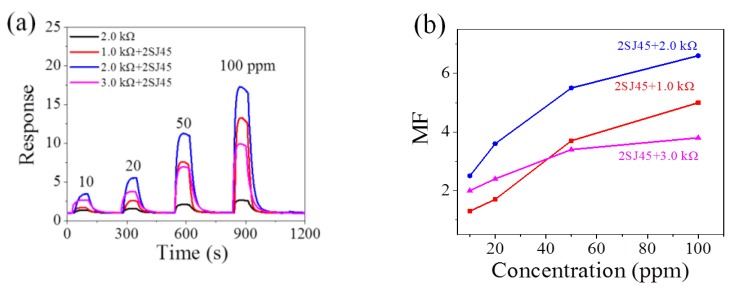
(**a**) Response of MP-4 to methane from 10 ppm to 100 ppm in the traditional electric circuit and the 2SJ45 FET circuit; (**b**) magnification factors of the 2SJ45 FET circuit (*R_L_* is 1.0, 2.0 and 3.0 kΩ respectively).

2SJ45 (~0.04 kΩ in the ON state) is adopted in the p-type FET amplification circuit (seen in Figure 1). *R_L_* is set as 1.0, 2.0, and 3.0 kΩ and the typical response curves with and without 2SJ45 FET are shown in Figure 2a and in Figure S3a in the supporting information. Figure 2a shows that the response to 100 ppm methane is about 2, in accordance with that of the manual, although there would be device–device variations and gas concentration variations. Figure 2a also illustrates that 2SJ45 is capable of enhancing the response of the MP-4 sensor to methane from 10 to 100 ppm to varying degrees. The resultant amplifications are shown in Figure 2b by testing at least four sensors, where it is obvious that a maximum *MF* of ~6.5 is obtained using the 2.0 kΩ resistor. In the meantime, it is noteworthy that every *MF* of the FET circuit with 2.0 kΩ is larger than that of the FET circuit with 1.0 and 3.0 kΩ because high *R_L_* would result in high (*R_L_* + *R_FET,a_*), while low *R_L_* would lead to low (*R_L_* + *R_FET,g_*) leading to low *MF* of (*R_L_* + *R_FET,__g_*)/(*R_L_* + *R_FET,__a_*) [20].

**Figure 3:**
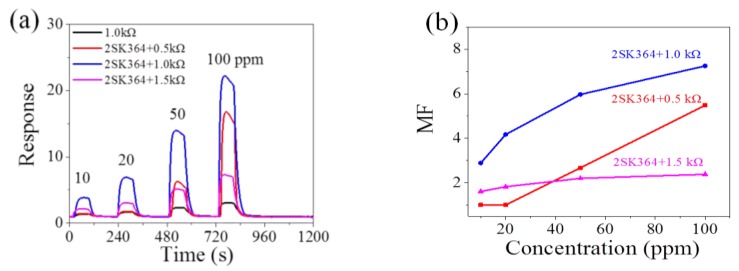
(**a**) Response of MP-4 to methane from 10 ppm to 100 ppm in the traditional electric circuit and the 2SK364 FET circuit; (**b**) magnification factors of the 2SK364 FET circuit (*R_L_* is 0.5, 1.0 and 1.5 kΩ respectively).

In our previous work, a single n-type FET amplification circuit with 2SK544 has amplified the signal of three kinds of MOX gas sensors (Figaro TGS2602 sensor for toluene, Hanwei MQ3 sensor for ethanol, Hanwei MP502 sensor for acetone) successfully [20]. In this paper, 2SK364 (~0.05 kΩ in the ON state) is exploited in the n-type FET amplification circuit (seen in Figure S2). Load resistance is set as 0.5, 1.0, and 1.5 kΩ, individually. Figure 3a shows that 2SK364 is able to significantly improve the response of the MP-4 sensor to methane (the corresponding output voltage versus time curves are seen in Figure S3b). Figure 3b illustrates that the magnification effect is maximum when *R_L_* is 1.0 kΩ with the magnification factor up to 7.3. The magnification factor (from 3 to 7.3) depends on the concentration of methane because different concentrations of methane generate different *V_GS_* change of FET, which in turn, leads to different resistance change.

**Figure 4:**
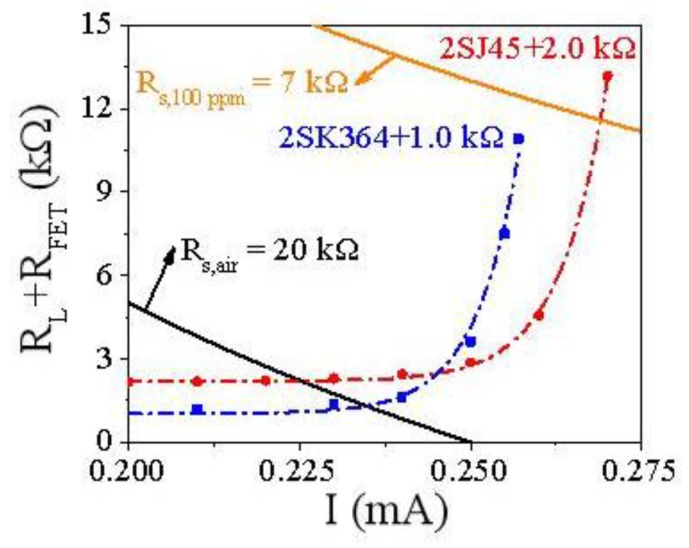
The scatter diagrams and fitting curves of the n-type FET 2SK364 circuit (blue dash curve) and the p-type FET 2SJ45 circuit (red dash curve) with *R_L_* 1.0 and 2.0 kΩ respectively.

As mentioned above, the 2SJ45 FET circuit with the 2.0 kΩ resistor and the 2SK364 FET circuit with the 1.0 kΩ resistor are the optimal amplification circuits. They both have different MFs in the same concentration of methane. So, it is essential to quantify their MFs in the given concentration of methane in theory. Thus, the principle of 2SK364 and 2SJ45 enhancing the response of the MP-4 sensor to methane has been further studied. Figure 4 shows the scatter diagrams and exponential function fitting curves of p-type and n-type FET circuits.

For the p-type FET circuit:

*R_L_ + R_p_ = (V_GS(p)_ + V_DS(p)_)/I* = 2.19 + 5.39 × 10^−17^ exp(147.60 × *I*) *r*^2^ = 0.999 (1)

For the n-type FET circuit:

*R_L_ + R_n_ = (V_GS(n)_ + V_DS(n)_)/I* = 1.03 + 2.53 × 10^−17^ exp(157.42 × *I*) *r*^2^ = 0.989 (2)

where *R_L_* is the load resistance; *R_p_* and *R_n_* are the resistances of 2SJ45 and 2SK364 respectively. *V_GS(p)_* and *V_DS(p)_* are *V_GS_* and *V_DS_* of 2SJ45 respectively. *V_GS(n)_* and *V_DS(n)_* are *V_GS_* and *V_DS_* of 2SK364 respectively. *I* represents the circuit current.

The black curve and orange solid curve in Figure 4 are expressed respectively as:

*R_L_ + R_FET,__air_ = V_CC_/I − R_S,air_* (3)

*R_L_ + R_FET,100ppm_ = V_CC_/I − R_S,100ppm_* (4)

where *V_CC_* is the applied voltage (generally 5 V), *R_FET,air_* and *R_FET,100ppm_* represent FET resistance in air and 100 ppm methane respectively. *R_S,air_* and *R_S,100ppm_* are the MP-4 sensor resistance (*R_S_*) in air and in 100 ppm methane individually. *R_S,air_* is 20 kΩ and *R_S,100ppm_* is 7 kΩ, determined experimentally.

The magnification factor (*MF*) of the single *FET* circuit is calculated by Formula (5) [20]:

*MF = (R_L_ + R_FET,100ppm_)/(R_L_ + R_FET,air_)* (5)

For the p-type FET circuit, the value of *R_L_* + *R_FET,air_* is the intersection y-coordinate of the Formulas (1) and (3), then *R_L_* + *R_FET,air_* is 2.2 kΩ. Similarly, *R_L_* + *R_FET,100ppm_* is the intersection y-coordinate of Formulas (1) and (4), then *R_L_* + *R_FET,100ppm_* is 11.59 kΩ. Therefore, the theoretical *MF* is calculated as 11.59 kΩ/2.20 kΩ = 5.3 with the actual *MF* of 6.5. In the same way, the theoretical *MF* of n-FET circuit can be calculated. However, Formulas (2) and (3) do not intersect with each other in Figure 4, which means that the amplification effect of n-FET 2SK364 is saturated in 100 ppm methane. Then, *R_L_*+ *R_FET,100ppm_* is 10.92 kΩ, i.e., the maximum of Formula (2). Finally, the theoretical *MF* is 10.92 kΩ/1.27 kΩ = 8.6 with the actual *MF* of 7.3. The theoretical and actual *MF*s are deemed to be consistent with each other.

### 4.2. Coupling the p+n FET Amplification Circuit

The experimental results above show that 1.0 kΩ and 2.0 kΩ are the optimal load resistance (*R_L_*) for n-type and p-type FET circuits respectively. Thus, a design scheme is put forward, so that 2SK364 and 2SJ45 are combined in the coupling p+n FET amplification circuit with *R_L_* of 1.0 kΩ, as shown in Figure 1. As mentioned above, when methane is injected into the gas chamber and because 1.0 kΩ is the optimal *R_L_* of 2SK364, not only is the output voltage amplified (the first *V_OUT_* amplification process) but also the resistance of 2SK364 rises. Once *R_L_* and 2SK364 resistance exceed 2.0 kΩ, 2SJ45 will realize the second *V_OUT_* amplification process. Therefore, this coupling p+n FET amplification circuit is theoretically feasible.

**Figure 5:**
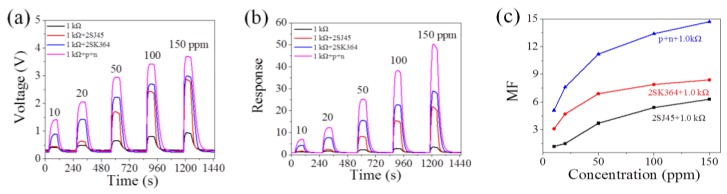
Amplification effect of the coupling p+n FET circuit. Comparison of (**a**) output voltage and (**b**) the response among 2SJ45, 2SK364, coupling p+n FET and FET free circuits by connecting a resistor of *R_L_* = 1.0 kΩ, and (**c**) magnification factors comparison among three FET circuits.

Figure 5a shows the transient *V_OUT_* versus time curves (each *R_L_* is 1.0 kΩ). When the MP-4 sensor responds to 10 ppm and 20 ppm methane, it is found that the single 2SJ45 does not significantly enhance *V_OUT_* (red line in Figure 5a), while the single 2SK364 (blue line in Figure 5a) obviously does. The highest *V_OUT_* belongs to the coupling p+n FET circuit (magenta line in Figure 5a); however, 2SJ45 in this coupling circuit plays a role in *V_OUT_* amplification, induced by the amplification effect of 2SK364. For higher concentrations of methane (≥50 ppm), these two FETs simultaneously play an excellent role in enhancing *V_OUT_*, as seen in Figure 5a.

Figure 5a,b shows that the MP-4 sensor in the coupling p+n FET circuit responds to 10 ppm with a response of 7.0 ± 0.2 and a voltage change of 1.1 ± 0.1 V, which is high enough to drive an alarmer, preventing the concentration of methane from 5%. At the same time, this coupling circuit extends the LOD of MP-4 from several hundred ppm to 10 ppm. Hence, this coupling circuit enables the MP-4 sensors to effectively detect low concentration methane. In addition, FETs required in this coupling circuit are easy to install and integrate and the cost decreases in bulk production. Figure 5c is the amplification factor versus the concentration curves. It is seen that the amplification factor of the coupling p+n FET circuit is much higher than that of the single FET circuit, with the maximum MF reaching 15. Figure S4 illustrates that the response of the MP-4 gas sensor in this coupling p+n circuit is exponential to the concentration, varying from 10 to 150 ppm, making it convenient to detect methane using this methodology in practical application.

**Figure 6:**
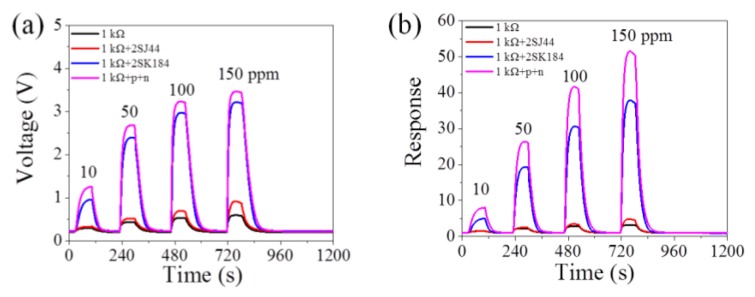
(**a**) Output voltage and (**b**) the response among 2SJ44, 2SK184, coupling p+n FET and FET free circuits by connecting a resistor of *R_L_* = 1.0 kΩ.

To further prove the universality of this coupling circuit, the p-type FET 2SJ44 and n-type FET 2SK184 are chosen to form a coupling circuit according to Figure 1. This coupling 2SJ44 + 2SK184 circuit also shows an excellent amplification effect. More detailed information is shown in Figure 6.

### 4.3. Mechanism of the Coupling p+n FET Amplification Circuit

**Figure 7:**
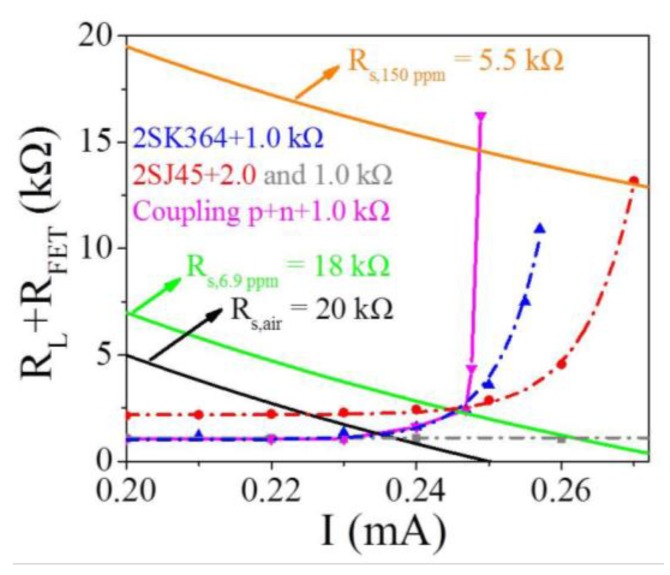
The approximate curve of the coupling p+n FET circuit with *R_L_* of 1.0 kΩ (magenta solid line). The scatter diagrams and fitting curves of the n-type FET 2SK364 circuit with *R_L_* 1.0 kΩ (blue dash curve) and p-type FET 2SJ45 circuits with *R_L_* 1.0 and 2.0 kΩ respectively (gray and red dashed lines).

The amplification effect of the coupling p+n FET circuit is then studied quantitatively. The blue dashed line in Figure 7 also represents the fitting curve of the n-type FET amplification circuit with 1.0 kΩ. The gray and red dashed lines represent the fitting curves of the p-type FET amplification circuits with *R_L_* of 1.0 and 2.0 kΩ, respectively. The black and orange solid curves in Figure 7 are similar to those in Figure 4. *R_S,air_* is 20 kΩ and *R_S,150 ppm_* is 5.5 kΩ, determined experimentally. For the green line, *R_S,__6.9ppm_* is 18 kΩ, determined theoretically, as shown in Figure S5.

According to the coupling p+n FET circuit in Figure 1 and Ohmic Law, the following formula can be obtained:

*V_GS(p)_ = V_GS(n)_ + V_DS(n)_,* (6)

*V_OUT_ = V_GS(p)_ + V_DS(p)_,* (7)

*R_L_ + R_FET_ = V_OUT_/I,* (8)

The formula of the working curve of the coupling p+n FET circuit can be obtained from Formulas (6–8).

*R_L_ + R_FET_ = (V_GS(n)_ + V_DS(n)_)/I + V_DS(p)_/I,* (9)

*(V_GS(n)_ + V_DS(n)_)/I* in Formula (9) is exactly the Formula (2) of the n-type FET circuit, reflecting the magnification effect of 2SK364. Meanwhile, *V_DS(p)_/I* in Formula (9) is only a part of Formula (1) of the p-type FET circuit. The *V_DS(p)_* is difficult to analyze because it is under the double effect of *V_GS_* of the two FETs. That is to say, the amplification effect of FET 2J45 is dependent on FET 2SK364. Therefore, the measurement of the working curve of the coupling p+n FET circuit needs to be simplified, approximated, and estimated, and can be divided into three stages:

- The first stage is in clean air, i.e., the baseline of the sensor. The *R_L_* of the coupling p+n FET circuit is 1.0 kΩ. Both of the FETs are in the ON state, so their resistances can be neglected. Therefore, the coupling circuit in clean air is equivalent to the single n-type or p-type FET circuit. Hence, the curves of both the coupling circuit (magenta solid line) and the single n- and p-type FET circuits (blue and gray dash lines) overlap each other, where *R_L_ + R_FET_* *≈ V_GS(n)_/I =* 1.0 kΩ, as seen in Figure 7.
- When trace concentrations (≤6.9 ppm) of methane gas are injected, which is the second stage, 2SK364 starts to enter the OFF state and amplifies *V_OUT_*, while 2SJ45 is still in the ON state. Therefore, the coupling p+n FET circuit is equivalent to the single n-type FET circuit, namely *R_L_* + *R_FET_* = (*V_GS(n)_* + *V_DS(n)_)/I*, whose curve overlaps the fitting curve of the single n-type FET circuit with *R_L_* of 1.0 kΩ (blue dash curve), as shown in Figure 7. It should be noted that all circuits, with or without FETs, cannot effectively detect methane lower than 6.9 ppm if the effective voltage signal is set to 1.0 V.
- Injecting more than 6.9 ppm of methane gas is the third stage. Both 2SK364 and 2SJ45 play an excellent amplifying role. In this stage, the coupling p+n FET circuit is roughly thought of as the single p-type FET circuit, where *R_L_* is subject to the n-FET. Consequently, this coupling circuit can be regarded as the p-type FET circuit with variable *R_L_*, which is distinguished from constant *R_L_* in the former single FET circuit and gives far higher *MF* than single FET circuits.

The p-type FET circuit with variable *R_L_* is then emphasized. The experimental results show that 2.0 kΩ is the optimal *R_L_*. Hence, 2.0 kΩ is set as the initial resistance. The fitting curves of the p-type FET circuit with constant *R_L_* of 2.1, 2.2 and 2.3 kΩ are shown in Figure S6. The selection of working points of the p-type FET circuit with variable R_L_ follows two requirements. One is that the point is obviously higher than the fitting curve of the n-type FET circuit (Formula (2)), as seen in Figure 7 to display the amplification role of *V_DS(p)_/I*, i.e., FET 2SJ45. The other is that the current of the selected point increases rather than decreases. According to these two requirements, the working curve of the coupling p+n FET circuit in the third stage is roughly estimated, which increases almost vertically, as seen in Figure 7. This is the basic reason why the coupling circuit is capable of amplifying the *V_OUT_* strikingly.

The working curve of this coupling p+n FET circuit is the magenta solid curve in Figure 7. It reveals the principle of this coupling circuit, that the n-FET firstly generates a *V_OUT_* amplification process and then p-FET continues based on the pre-amplification process, where *V_OUT_* amplification is derived from the gate voltage inducing resistance change of the FET. It is noteworthy that the estimated curve of this coupling circuit is not the simple superposition of two fitting curves of the p-type and the n-type FET circuits, but rather, a more complex combination. According to Figure 7, the theoretical magnification factor (*MF*) is calculated as 14.61 kΩ/1.07 kΩ = 13.7, which is the actual *MF* of 15 in Figure 5c. This indirectly proves the reliability of the estimated curve of the coupling p+n FET circuit.

Moreover, it is also noted that the response time of 15–18 s is not affected by the FETs and neither is the recovery time, as shown in Table S1. Finally, this methodology can also be adopted to detect other gases, as the amplification depends only on the FETs, not on the gases. This is similar to the n-type 2SK544 FET circuit, which is able to amplify the signal of the commercial toluene, ethanol, and acetone MOX gas sensors [20].

For contrast, 2SJ45 and 2SK364 are combined in a circuit forming the former synergetic p+n FET circuit [28] in Figure S7. Obviously, the gate of the p-FET is not connected to the drain of the n-FET but the higher potential side of *R_L_*, which distinguishes the synergetic circuit from the coupling circuit. It should be noted that the p-type and the n-type FETs in this synergetic circuit can work independently. Accordingly, the working curve of the synergetic p+n FET circuit is expressed as below:

*R_L_ + R_FET_ = (V_GS(n)_ + V_DS(n)_)/I + (V_GS(p)_ + V_DS(p)_)/I* – 1 kΩ, (10)

Apparently, the curve of this synergetic circuit shown in Figure S8 is the simple superposition of two working curves of the p-type and n-type FET circuits. Figure S8 also illustrates that the synergetic 2SJ45 + 2SK364 circuit has a similar working curve with a single 2SK364 circuit. Thus, it is theoretically infeasible to combine 2SJ45 and 2SK364 in this synergetic p+n FET circuit.

## 5. Conclusions

In this paper, a novel coupling FET circuit is designed to detect low concentrations methane. The commercial sensor (MP-4) used in this coupling circuit has shown a response of 7.0 ± 0.2 to 10 ppm methane. Further, the limit of detection decreases from several hundred ppm to 10 ppm, extended by an order of magnitude. It is expected that the monitoring of methane leakage and the early warning of methane explosions will be achieved. At the same time, this coupling FET circuit can amplify the response to methane by 15 times, with the response time and recovery time unaffected by FETs. More importantly, FETs used in this coupling circuit are easy to install, integrate, and the cost increases by only ~10% of the sensor by adding two FETs. The mechanism of this coupling circuit is that the n-FET firstly generates an output voltage (*V_OUT_*) amplification process, caused by the gate voltage-induced resistance change of the FET. Then, the p-FET continues based on the previous *V_OUT_* amplification process. It is a more complex combination rather than the simple superposition of two fitting curves of p-type and n-type FET circuits. Finally, this coupling circuit is also promising in other kinds of MOX sensors to detect low concentrations of the target gases.

## Supplementary Materials

The following are available online at http://www.mdpi.com/1424-8220/18/3/787/s1, Figure S1: (a) I_DS_–V_GS_ curve of four p-type FETs. (b) I_DS_–V_GS_ curve of four n-type FETs. (c) dR_DS_/dV_GS_-V_GS_ curve of 2SJ45 (red) and 2SK364 (blue), Figure S2: Design scheme of the conventional electric circuit, designed single n-type FET amplification circuit for MOX methane sensors, Figure S3: (a) Output voltage of MP-4 to methane from 10 to 100 ppm in the traditional electric circuit and the 2SK364 FET circuit (R_L_ is 0.5, 1.0 and 1.5 kΩ respectively). (b) Output voltage of MP-4 to methane from 10 ppm to 100 ppm in the traditional electric circuit and the 2SJ45 FET circuit (R_L_ is 1.0, 2.0 and 3.0 kΩ respectively), Figure S5: Linear fitting line of response of MP-4 versus methane concentration from 10 to 50 ppm in the traditional electric circuit with R_L_ of 1.0 kΩ (Linear fitting line is Res = 1.00 + 0.016c, *r*^2^ = 0.9996), Figure S6: The approximate curve of the coupling p+n FET circuit with R_L_ 1.0 kΩ (magenta solid curve). The scatter diagrams and fitting curves of the n-type FET 2SK364 circuit with R_L_ 1.0 kΩ (blue dash curve) and p-type FET 2SJ45 circuits with R_L_ 1.0, 2.0, 2.1, 2.2 and 2.3 kΩ respectively, Figure S7: Design scheme of the synergetic p+n amplification circuit for the MOX methane gas sensor, Figure S8: The working curve of the synergetic p-type 2SJ45 and n-type 2SK364 circuit with R_L_ of 1.0 kΩ, Table S1: The response time of gas sensor in different circuits with or without FET.
