# Supplementary material for: Coupling p+n Field-Effect Transistor Circuits for Low Concentration Methane Gas Detection
Source: Sensors (Basel). 2018 Mar 6;18(3):787. doi: 10.3390/s18030787 (PMC5876617; doi:10.3390/s18030787)
Supplement: Supplementary file 1 [file sensors-18-00787-s001.doc]

Article

Supplementary Materials: Coupling p+n Field-effect Transistor Circuits for Low Concentration Methane Gas Detection

Xinyuan Zhou^1,2^, Liping Yang^1^, Yuzhi Bian^1,2^, Xiang Ma^1,2^, Ning Han^1,3^,* and Yunfa Chen^1,3^,*

^1^ State Key Laboratory of Multiphase Complex Systems, Institute of Process Engineering, Chinese Academy of Sciences, Beijing 100190, China

^2^ University of Chinese Academy of Sciences, No. 19A Yuquan Road, Beijing 100049, China zhouxinyuan14@mails.ucas.edu.cn

^3^ Center for Excellence in Regional Atmospheric Environment, Institute of Urban Environment, Chinese Academy of Sciences, Xiamen 361021, China

***** Correspondence: nhan@ipe.ac.cn; chenyf@ipe.ac.cn; Tel.: +86-010-6255-8356

| 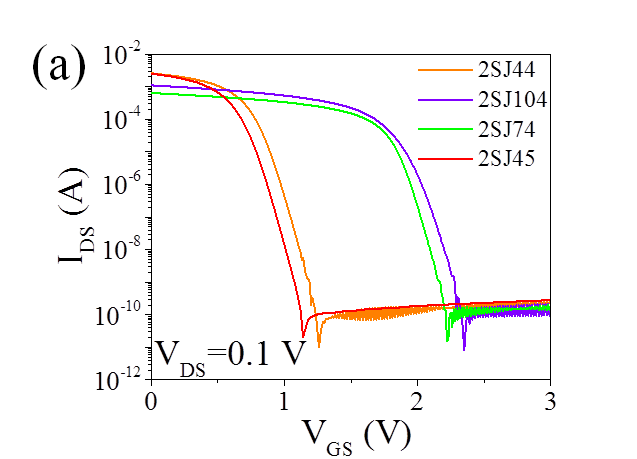 | 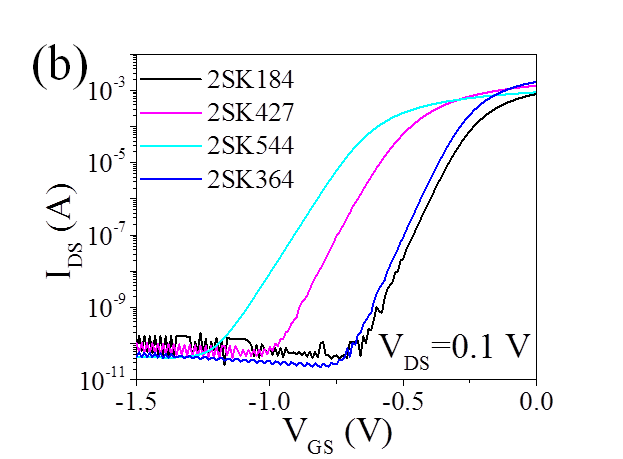 | 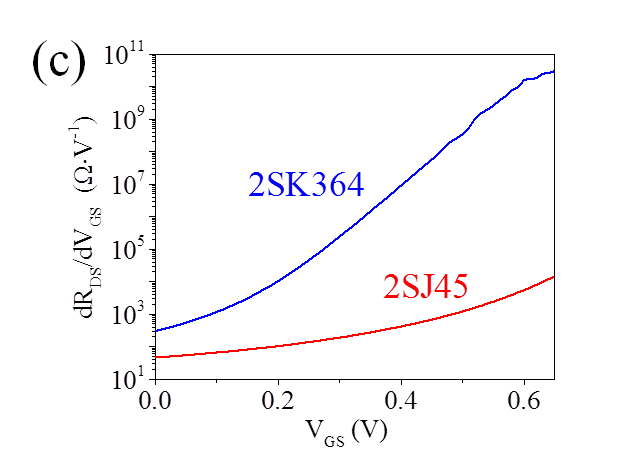 |
| --- | --- | --- |

**Figure S1.** (a) I_DS_-V_GS_ curve of four p-type FETs. (b) I_DS_-V_GS_ curve of four n-type FETs. (c) dR_DS_/dV_GS_-V_GS_ curve of 2SJ45 (red) and 2SK364 (blue).

| 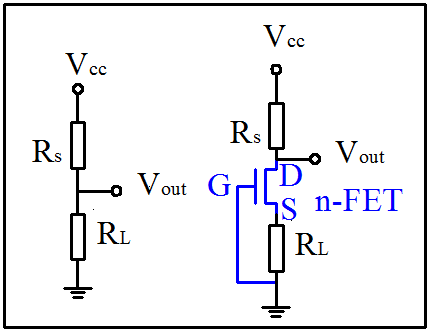 |
| --- |

**Figure S2.** Design scheme of the conventional electric circuit, designed single n-FET amplification circuit for MOX methane sensors.

| 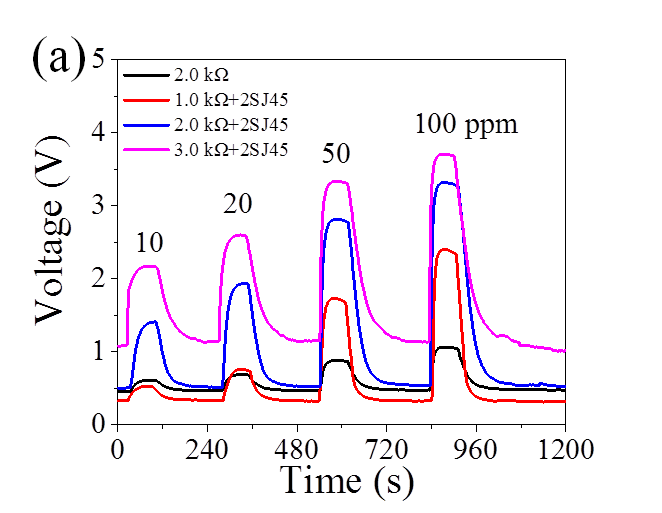 | 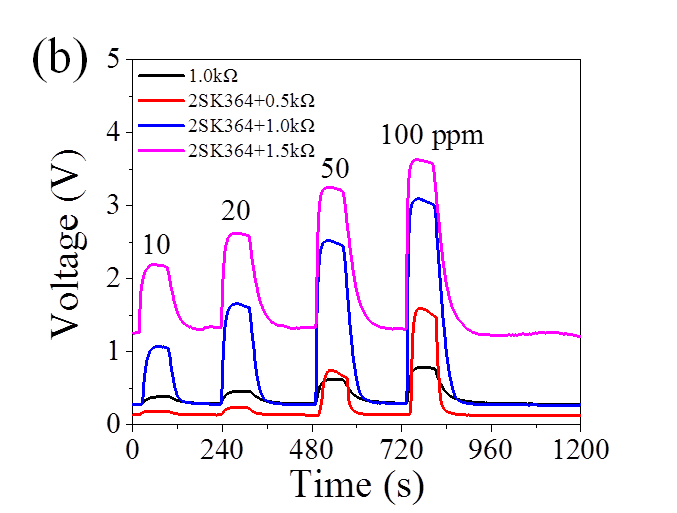 |
| --- | --- |

**Figure S3.** (a) Output voltage of MP-4 to methane from 10 ppm to 100 ppm in the traditional electric circuit and the 2SJ45 FET one (R_L_ is 1.0, 2.0 and 3.0 kΩ respectively). (b) Output voltage of MP-4 to methane from 10 to 100 ppm in the traditional electric circuit and the 2SK364 FET one (R_L_ is 0.5, 1.0 and 1.5 kΩ respectively).


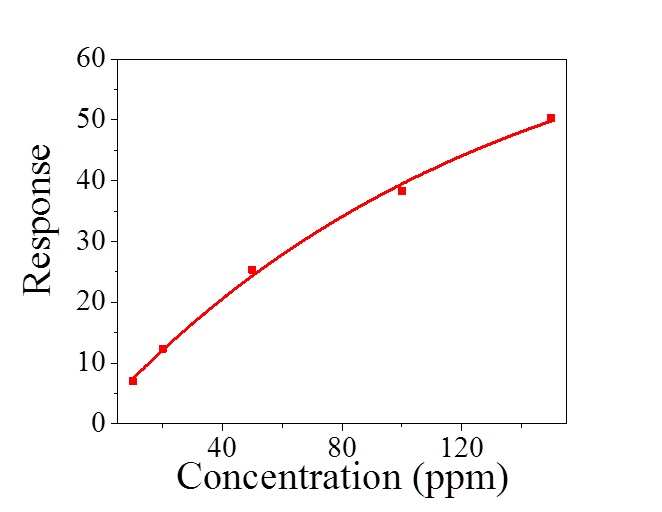


**Figure S4.** The exponential fitting curve of response of MP-4 in coupling p+n FET circuit with R_L_ of 1.0 kΩ. (Res = 71.17 - 70.16 exp(0.0755 c), r^2^=0.996 ).

| 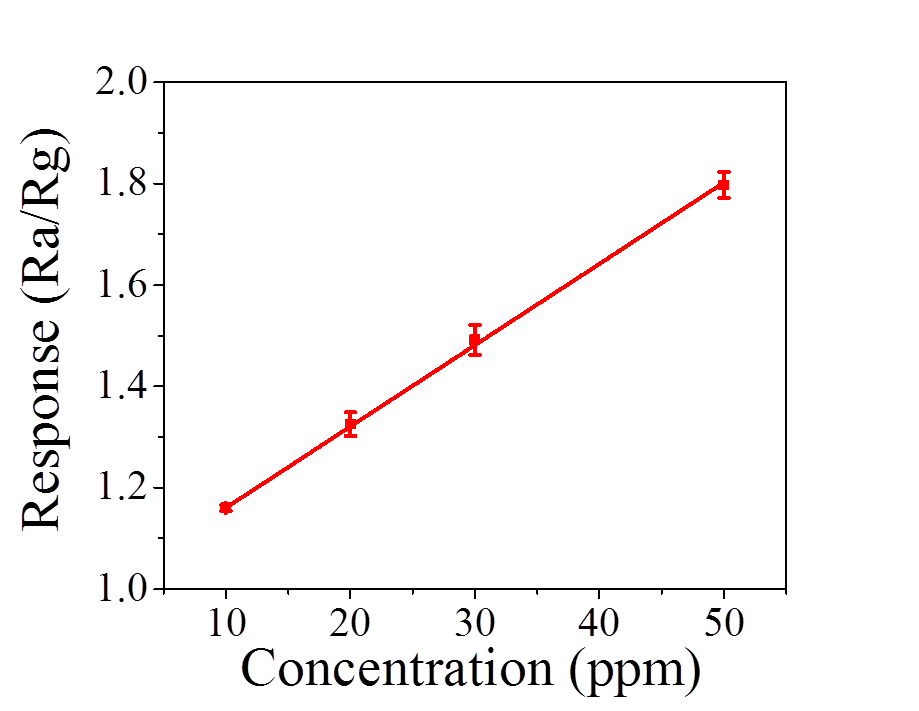 |
| --- |

**Figure S5.** Linear fitting of response of MP-4 versus methane concentration from 10 to 50 ppm in the traditional electric with R_L_ of 1.0 kΩ (Linear fitting is Res = 1.00 + 0.016c, r^2^ = 0.9996).

| 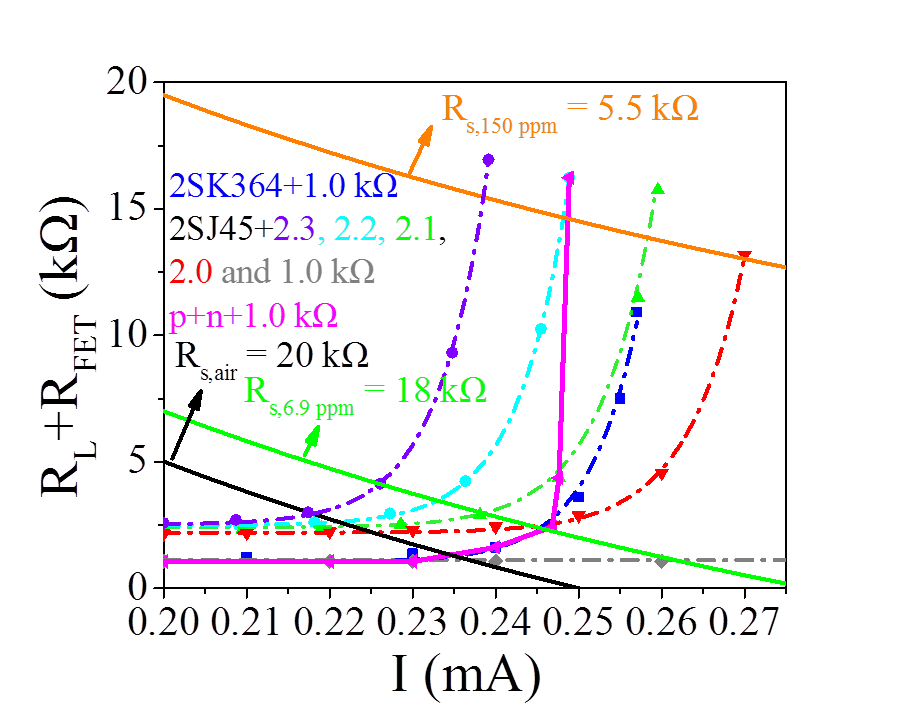 |
| --- |

**Figure S6.** The approximate curve of coupling p+n FET circuit with R_L_ 1.0 kΩ (magenta solid curve). The scatter diagrams and fitting curves of n-type FET 2SK364 circuit with R_L_ 1.0 kΩ (blue dash curve) and p-type FET 2SJ45 circuits with R_L_ 1.0, 2.0, 2.1, 2.2 and 2.3 kΩ respectively.

| 1.0 kΩ: R_L_ + R_p_ = 1.12 + 3.02 × 10^-16^exp(75.37 × *I* )  r^2^=0.999, | (S1) |
| --- | --- |
| 2.0 kΩ: R_L_ + R_p_ = 2.19 + 5.39 × 10^-17^exp(147.60 × *I*)  r^2^=0.999, | (S2) |
| 2.1 kΩ: R_L_ + R_p_ = 2.31 + 1.19 × 10^-16^exp(151.26 × *I* )  r^2^=0.9995, | (S3) |
| 2.2 kΩ: R_L_ + R_p_ = 2.49 + 1.65 × 10^-17^exp(165.81 × *I* )  r^2^=0.9999, | (S4) |
| 2.3 kΩ: R_L_ + R_p_ = 2.52 + 8.09 × 10^-17^exp(166.09 × *I* )  r^2^=0.9996, | (S5) |
| 1.0 kΩ: R_L_ + R_n_ = 1.03 + 2.53 × 10^-17^exp(157.42 × I)  r^2^ = 0.989, | (S6) |

| 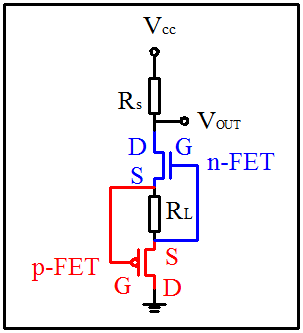 |
| --- |

**Figure S7.** Design scheme of the synergetic p+n amplification circuit for MOX methane gas sensor.

| 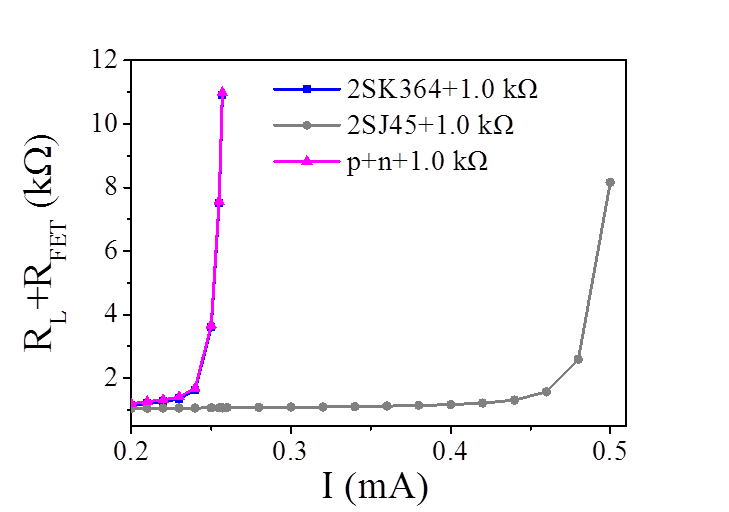 |
| --- |

**Figure S8.** The working curve of the synergetic p-type 2SJ45 and n-type 2SK364 circuit with R_L_ of 1.0 kΩ.

**Table S1.** The response time of gas sensor in different circuits with or without FET.

| **50 ppm** | **Response time (s)** |
| --- | --- |
| 1 kΩ | 17 |
| 1 kΩ + 2SJ45 | 17 |
| 1 kΩ + 2SJK364 | 18 |
| 1 kΩ + 2SJ45 + 2SK364 | 18 |
| **100 ppm** | **Response time (s)** |
| 1 kΩ | 16 |
| 1 kΩ + 2SJ45 | 15 |
| 1 kΩ + 2SJK364 | 17 |
| 1 kΩ + 2SJ45 + 2SK364 | 17 |
| **150 ppm** | **Response time (s)** |
| 1 kΩ | 16 |
| 1 kΩ + 2SJ45 | 16 |
| 1 kΩ + 2SJK364 | 15 |
| 1 kΩ + 2SJ45 + 2SK364 | 16 |
